# A Simple Enzymatic Method for Production of a Wide Variety of D-Amino Acids Using L-Amino Acid Oxidase from *Rhodococcus* sp. AIU Z-35-1

**DOI:** 10.4061/2010/567210

**Published:** 2010-08-05

**Authors:** Kimiyasu Isobe, Hiroshi Tamauchi, Ken-ichi Fuhshuku, Shouko Nagasawa, Yasuhisa Asano

**Affiliations:** ^1^Department of Biological Chemistry and Food Science, Faculty of Agriculture, Iwate University, 3-18-8 Ueda, Morioka 020-8550, Japan; ^2^Biotechnology Research Center and Department of Biotechnology, Toyama Prefectural University, 5180 Kurokawa, Imizu, Toyama 939-0398, Japan

## Abstract

A simple enzymatic method for production of a wide variety of D-amino acids was developed by kinetic resolution of DL-amino acids using L-amino acid oxidase (L-AAO) with broad substrate specificity from *Rhodococcus* sp. AIU Z-35-1. The optimum pH of the L-AAO reaction was classified into three groups depending on the L-amino acids as substrate, and their respective activities between pH 5.5 and 8.5 accounted for more than 60% of the optimum activity. The enzyme was stable in the range from pH 6.0 to 8.0, and approximately 80% of the enzyme activity remained after incubation at 40°C for 60 min at pH 7.0. D-Amino acids such as D-citrulline, D-glutamine, D-homoserine or D-arginine, which are not produced by D-aminoacylases or D-hydantoinases, were produced from the racemic mixture within a 24-hr reaction at 30°C and pH 7.0. Thus, the present method using L-AAO was versatile for production of a wide variety of D-amino acids.

## 1. Introduction

d-Amino acids are useful raw materials for production of pharmaceuticals and agrochemicals. At present, some d-amino acids are produced by enzymatic methods on an industrial scale. For example, *p*-hydroxy-d-phenylglycine and d-phenylglycine are produced by the combination of d-hydantoinase and *N*-carbamyl-d-amino acid amidohydrolase [1–3]. d-Leucine, d-phenylalanine and d-methionine can be produced by an enzymatic resolution of* N*-acetyl-dl-leucine, *N*-acetyl-dl-phenylalanine and *N*-acetyl-dl-methionine, respectively, using bacterial *N*-acyl-d-amino acid deacylase (EC 3.5.1.81, d-aminoacylase) [4–6]. d-Valine is also produced by an enzymatic resolution of* N*-acetyl-dl-valine with d-aminoacylase from* Defluvibacter *sp. A131-3 [7]. Methods for the production of d-alanine, d-methionine, d-norvaline, d-norleucine or d-phenylalanine were demonstrated using new enzymes such as d-stereospecific aminopeptidases (EC 3.4.11.19, d-aminopeptidase) [8] or d-stereoselective amino acid amidases [9–11]. The production methods of d-glutamic acid, d-proline or d-homoserine were also demonstrated using selective degradation of the corresponding l-isomer in a racemic mixture [12–14]. These enzymatic methods are useful tools for production of some d-amino acids, but not always useful for production of a wide variety of d-amino acids because of their limited substrate specificity. Recently, we isolated a new bacterial strain, *Rhodococcus* sp. AIU Z-35-1, for production of *N*α**-benzyloxycarbonyl-l-aminoadipic acid (*N*α**-Z-l-AAA) from *N*α**-benzyloxycarbonyl-l-lysine (*N*α**-Z-l-lysine) [15]. In the analysis of this reaction, it was demonstrated that *N*α**-Z-l-lysine was converted to *N*α**-Z-l-AAA via *N*α**-benzyloxycarbonyl-l-aminoadipate-*δ*-semialdehyde (*N*α**-Z-l-AASA), and the conversion of *N*α**-Z-l-lysine into* N*α**-Z-l-AASA was catalyzed by a l-specific amino acid oxidase (l-AAO) with broad substrate specificity [16]. Thus, the l-AAO catalyzed an oxidative deamination of *N*α**-acyl-l-lysine, *N*ε**-acyl-l-lysine, l-lysine and many other l-amino acids, but not of *N*α**-acyl-d-lysine, *N*ε**-acyl-d-lysine and d-amino acids. These results indicate that l-AAO produced by *Rhodococcus* sp. AIU Z-35-1 may be applicable for production of a wide variety of d-amino acids from dl-amino acids by kinetic resolution. The present paper describes an enzymatic method for production of d-amino acids, which are difficult to produce by the d-aminoacylases, d-hydantoinases or d-amino acid amidases, using l-AAO from *Rhodococcus* sp. AIU Z-35-1.

## 2. Materials and Methods

### 2.1. Chemicals

*N*α**-Z-l-lysine and *N*α**-Z-d-lysine were purchased from Watanabe Chemical Industries (Hiroshima, Japan). d- and l-Glutamine, d- and l-arginine, d- and l-citrulline, d- and l-homoserine and other amino acids were from Wako Pure Chemicals (Osaka, Japan). All other chemicals used were of analytical grade and were purchased commercially. The l-AAO from *Rhodoccocus *sp. Z-35-1 was purified according to our method described in a previous report [16].

### 2.2. Assay of Enzyme Activity

l-AAO activity was assayed by measuring the rate of hydrogen peroxide formation as follows. The standard reaction mixture contained 40 *μ*mol of l-lysine, 0.6 *μ*mol of 4-aminoantipyrine, 1.94 *μ*mol of *N*-ethyl-*N*-(2-hydroxy-3-sulfopropyl)-3-methylaniline sodium salt dihydrate, 6.7 units of peroxidase, 0.1 mmol of potassium phosphate, pH 7.0, and an appropriate amount of enzyme, in a final volume of 1.0 ml. The hydrogen peroxide formation was spectrophotometrically followed at 30°C by measuring absorbance at 555 nm. One unit (U) is the amount of enzyme that catalyzes the formation of one micromole of hydrogen peroxide from l-lysine per min. 

### 2.3. Assay of Optimum pH and pH Stability

Optimum pH of l-AAO activity was analyzed under standard assay conditions, except that the reaction pH values were varied between 5.0 and 8.5 by 0.1 mmol of acetate buffer, pH 5.0–5.5 or 0.1 mmol of potassium phosphate, pH 5.5–8.5. The pH stability of l-AAO was analyzed by assaying the residual activity after incubation with 50 mM potassium phosphate, pH 5.5–8.5, at 40°C for 60 min. The thermal stability of l-AAO was analyzed by assaying the residual activity after incubation with 50 mM potassium phosphate, pH 7.0, at 30–60°C for 60 min. 

### 2.4. Standard Reaction of l-Amino Acid Oxidation and d-Amino Acid Production

l-Amino acid oxidation was carried out by incubation of 20 micromoles of l-amino acids with 15.5 × 10^−3^ U of l-AAO at 30°C for 24 hr in 1 ml of 0.1 M potassium phosphate buffer, pH 7.0, containing 1700 units of catalase with shaking at 120 rpm. Production of d-amino acids from dl-amino acids was carried out under the same conditions as the l-amino acid oxidation using 100 micromoles of dl-amino acids in 1 ml of reaction mixture. 

### 2.5. Determination of d- and l-Amino Acids and Reaction Product of l-Amino Acid Oxidation

When l-amino acids were used as substrate, the reaction products were separated from unconverted l-amino acids by HPLC with a TSKgel DEAE-5PW column (Tosoh, Tokyo, Japan) at a flow rate of 0.8 ml per min at 40°C. In this HPLC analysis, the products were eluted with water for 5 min, followed by increasing the NaCl concentration to 0.3 M with a linear gradient for 10 min, and then by 0.3 M NaCl for 10 min. Detection was carried out at 210 nm, and the product amounts were calculated from the peak area.

When a mixture of d- and l-amino acids was used as substrate, product from l-amino acid oxidation was first separated from d-amino acid and unconverted l-amino acid by HPLC with a TSKgel DEAE-5PW column under the same conditions as above. The fraction containing d- and l-amino acids was then applied to HPLC with a CROWNPAK CR(+) (Daicel Chemical Industries, Tokyo, Japan). This HPLC analysis was carried out at a flow rate of 0.5 ml/min at 25°C using a HClO_4_ solution, pH 1.0–2.0. Detection was carried out at 200 nm, and amounts of d-amino acid and unconverted l-amino acid were calculated from the peak area from HPLC. 

### 2.6. Identification of Reaction Product from l-Amino Acid Oxidation

Each reaction product from l-amino acids oxidation, which was obtained by HPLC with a TSKgel DEAE-5PW column, was concentrated by a SpeedVac concentrator, and then applied to HPLC with a CynProPep C18 column (Shimadzu, Kyoto, Japan). The eluate was concentrated again by a SpeedVac concentrator and applied to an HCT Ultra mass spectrometric instrument (Bruker Daltonics GmbH, Bremen, Germany). Sample was identified by electrospray ionization (ESI-) mass spectrometry (MS) or ESI-MS/MS. The electrospray ionization voltage of the capillary was set to −4500 V and the end plate to −500 V. Nitrogen was used as dry gas at a temperature of 300°C and a flow rate of 10 l/min. MS and MS/MS spectra were collected over a m/z range of 50–300 at 8,100 m/zs-1.

## 3. Results

### 3.1. Effects of pH on l-Amino Acid Oxidation and Enzyme Stability

**Figure 1:**
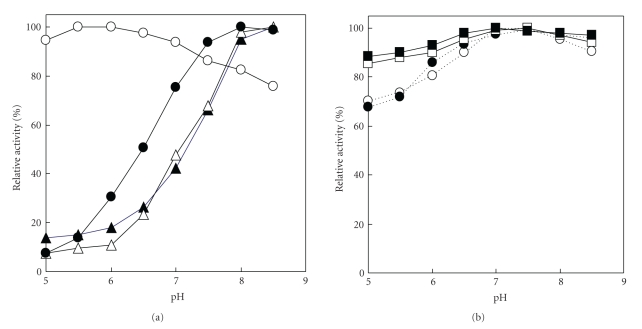
Effect of reaction pH on l-amino acid oxidation. The reaction was carried out under standard reaction conditions, except that the reaction pH values were varied between pH 5.0 and 8.5, using 0.1 mmol of acetate buffer, pH 5.0–5.5 and 0.1 mmol of potassium phosphate, pH 5.5–8.5. The following l-amino acids were used as substrate. (a) Open circles, l-lysine; closed circles, *N*α**-Z-l-lysine; open triangles, l-aspartic acid; closed triangles, l-glutamic acid. (b) Open squares (solid line), l-leucine; closed squares (solid line), l-ornithine; open circles (dotted line), l-asparagine; closed circles (dotted line), l-homoserine.

Effects of pH on l-amino acid oxidation were assayed using 2.21 × 10^−3^ U of l-AAO under standard assay conditions of enzyme activity measurement, except that the reaction pH values and l-amino acids were varied. The optimum pH of oxidase activity for acidic amino acids such as l-glutamic acid and l-aspartic acid ranged 8.0–8.5, and the activity at pH 6.0 was less than 30% of that at pH 8.5. These results were similar to those of* N*α**-Z-l-lysine. In contrast, the optimum pH of oxidase activity for l-lysine and all other l-amino acids tested was pH 5.5–6.0 and pH 7.0–7.5, respectively, and their respective activities between pH 5.5 and 8.5 accounted for more than 60% of the optimum activity (Figure 1). 

**Figure 2:**
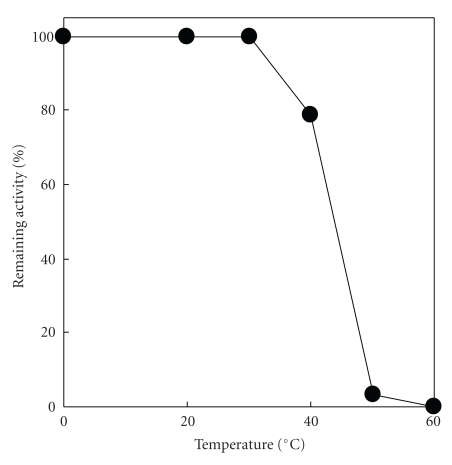
Effect of temperature on enzyme stability. The enzyme was incubated at 30–60°C for 60 min at pH 7.0, and its residual activity was assayed under standard assay condition of enzyme activity.

To analyze its pH stability, l-AAO was incubated at 40°C for 60 min in the pH region between 5.0 and 8.5, and the residual activities were assayed. The enzyme was stable in the pH region between 6.0 and 8.0 (data not shown). When this enzyme was incubated at 30–60°C for 60 min at pH 7.0, approximately 80% of the enzyme activity remained at 40°C, although the enzyme activity was almost completely lost at 50°C (Figure 2). On the basis of the optimum reaction pH and pH stability, the following studies were carried out at pH 7.0 and 30°C.

### 3.2. Identification of Products from l-Amino Acid Oxidation

Each l-amino acid indicated in Table 1 was incubated with 15.5 × 10^−3^ U of l-AAO under standard conditions of l-amino acid oxidation, and the reaction mixture was applied to HPLC with a TSKgel DEAE-5PW column. In this HPLC analysis, a new single peak was obtained from l-homoserine, l-leucine, l-lysine, l-glutamine, l-arginine, l-glutamic acid, l-phenylalanine, l-histidine and *N* *ε*-acetyl-l-lysine (Table 1), and the keto-group was confirmed in the compound of each peak. In addition, the molecular mass of the peak compounds was coincident with the theoretical value of *α*-keto-acid corresponding to the l-amino acid. In the case of l-citrulline, two peaks were newly obtained by HPLC with a TSKgel DEAE-5PW column (Table 1). When the compound of each peak was applied again to the same column, two peaks were also obtained from each compound at the same elution time, indicating that the oxidative products from l-citrulline are in a state of equilibrium. The molecular mass of both compounds was also coincident with the theoretical value of the corresponding *α*-keto-acid of l-citrulline (Table 1). Thus, it was presumed that one product from l-citrulline is 2-oxo-5-ureidovaleric acid and the other is pyrrolidine-1-carbamyl-2-hydroxy-2-carboxylic acid. These results experimentally supported our previous report that l-AAO from *Rhodococcus* Z-35-1 is l-specific amino acid oxidase with broad substrate specificity [16]. They also indicate that the above l-amino acids were converted to the corresponding *α*-keto-acids by the oxidative deamination of the *α*-amino group of l-amino acids.

### 3.3. l-Amino Acid Oxidation

Since enantioselective production of d-amino acids from dl-amino acids is achieved by the complete oxidation of l-amino acids, we investigated the optimum enzyme amounts for complete oxidation of l-amino acids under standard conditions using l-amino acids which are not substrates of d-aminoacylases, d-hydantoinases and d-amino acid amidases. When 20 mM of l-citrulline, l-glutamine, l-homoserine, l-leucine or *N*ε**-acetyl-l-lysine was incubated with 15.5 × 10^−3^ U of l-AAO at pH 7.0 and 30°C, those amino acids were completely converted to the corresponding *α*-keto-acids within 24 hr (data not shown). In the respective case of l-arginine, l-glutamic acid, l-phenylalanine, and l-histidine, 95%, 87%, 85% and 79% of the original amounts were converted to the corresponding *α*-keto-acids, respectively, under the same conditions (data not shown). The enzyme amounts for complete oxidation of 50 mM l-amino acids were further analyzed. l-Citrulline, l-homoserine, l-glutamine, l-leucine or *N*ε**-acetyl-l-lysine required 45 × 10^−3^ U of l-AAO for complete oxidation within 24 hr. For l-arginine, l-glutamic acid, l-phenylalanine or l-histidine, 60 × 10^−3^ U of l-AAO were required for complete oxidation within 24 hr. These results indicate that high concentration of l-amino acids and the reaction products do not inhibit the l-amino acid oxidation. They also indicate that enzyme amounts for complete oxidation of l-amino acids might be calculated in light of the present results and the relative reaction rate shown in [16].

### 3.4. Enzymatic Resolution of dl-Amino Acids

**Figure 3:**
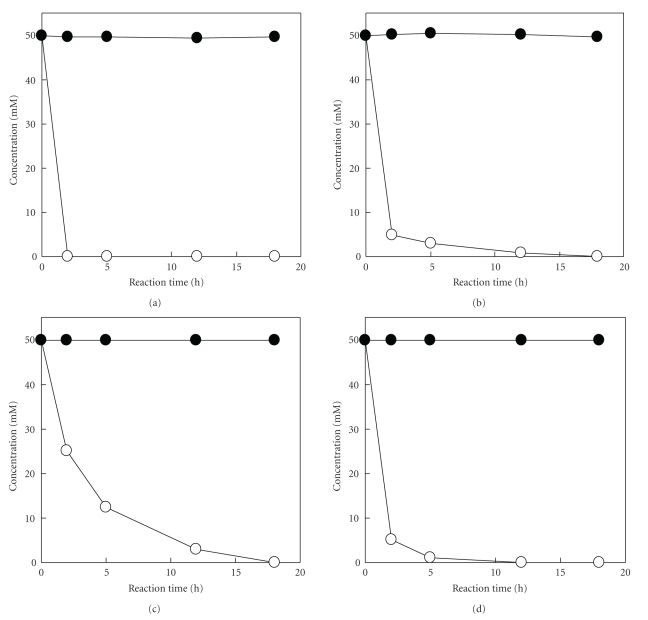
Production of d-amino acid. Approximately 100 mM of dl-homoserine (a), dl-glutamine (b), dl-citrulline (c) or *N*ε**-acetyl-dl-lysine (d) was incubated at 30°C for 24 hr under standard condition of d-amino acid production using 40 × 10^−3^ U of l-AAO. dl-Amino acids of reaction mixture were first separated from the reaction products by HPLC with a TSKgel DEAE-5PW column, and each d- and l-amino acid amount was analyzed by HPLC with a CROWNPAK CR(+) according to the method described in Section 2. Closed circles, d-amino acids; open circles, l-amino acids.

On the basis of the above results of the complete oxidation of l-amino acids, d-amino acid production was investigated using dl-amino acids as substrate. When 100 mM each of dl-citrulline, dl-glutamine, dl-homoserine or *N*ε**-acetyl-dl-lysine was incubated with 40 × 10^−3^ U of l-AAO, the l-amino acids were oxidized to the corresponding *α*-keto-acids within 24 hr of incubation, and d-amino acids remained without any reaction (Figure 3). When 100 mM dl-arginine was incubated with 60 × 10^−3^ U of l-AAO, l-arginine was also completely oxidized, but d-arginine was not (data not shown).

## 4. Discussion

At present, a wide variety of d-amino acids cannot be produced by one enzyme system, because presently available enzymes have a limited substrate specificity. Recently, we found a new l-AAO from *Rhodococcus *sp. AIU Z-35-1, which catalyzed oxidative deamination of constituent and nonconstituent l-amino acids of natural proteins. Therefore, we developed a new simple enzymatic method for production of a wide variety of d-amino acids from a racemic mixture by kinetic resolution using l-AAO from *Rhodococcus *sp. AIU Z-35-1. Firstly, we analyzed the optimum conditions for l-amino acid oxidation. When the *α*-amino group of l-lysine was covered with the benzyloxicarbonyl-group, optimum pH changed to 8.0–8.5. This pH range is similar to that of acidic amino acids such as l-glutamic acid and l-aspartic acid, suggesting that the optimum pH of l-amino acid oxidation is affected by the amino group of l-amino acids. Our studies of optimum pH also showed that the enzyme activity at pH 7.0 accounts for more than 60% of the optimum activity in l-amino acid oxidation tested. In addition, the enzyme was stable at pH 7.0. Therefore, we concluded that incubation at pH 7.0 is optimum for d-amino acid production by kinetic resolution using our l-AAO. Under the optimum conditions, some d-amino acids, which are difficult to produce by d-aminoacylases, d-hydantoinases or d-amino acid amidases, are efficiently produced, since high concentration of d-amino acids, l-amino acids and the reaction products do not inhibit the l-amino acid oxidation, although two oxidative products in the equilibrium state are produced from l-citrulline. Since it has been already demonstrated that l-AAO from *Rhodococcus *sp. AIU Z-35-1 is specific to l-amino acids and has broad substrate specificity [16], the present enzymatic method is also useful for production of other d-amino acids. 

The enzymatic method developed here has an advantage in that a wide variety of d-amino acids can be produced, although it also has a disadvantage in that the maximum yield of d-amino acids is theoretically 50% of the dl-amino acids used as a substrate. A further advantage of the new enzymatic method is that it is applicable for the production of keto-acids corresponding to l-amino acids used as substrate. Geueke and Hummel have reported that *R. opacus* DSM 43250 produced a new bacterial l-AAO with broad substrate specificity [17]. However, l-AAO of *R. opacus* is not advantageous for production of a high concentration of d-amino acids, since the enzyme was strongly inhibited by a low concentration of l-amino acids. The l-amino acid deaminases also catalyze the conversion of l-amino acid to the corresponding *α*-keto acids without formation of H_2_O_2_. Therefore, the l-amino acid deaminases are also useful for enantioselective production of d-amino acids from a racemic mixture. However, the substrate specificity of the l-amino acid deaminases reported [18, 19] is narrow compared to that of l-AAO from *Rhodococcus* sp. Z-35-1. Thus, l-AAO from *Rhodococcus* sp. Z-35-1 is the most versatile for enzymatic production of a wide variety of d-amino acids or keto-acids corresponding to l-amino acids from dl-amino acids.

## Figures and Tables

**Table 1:** Analysis of oxidation products from l-amino acids. Twenty micromoles of each l-amino acid was incubated with 15.5 × 10^−3^ U of l-AAO under standard conditions of l-amino acid oxidation, and the reaction mixture was applied to HPLC with a TSKgel DEAE-5PW column. Molecular mass of reaction products was analyzed by an HCT Ultra mass spectrometric instrument.

l-Amino acid	Elution time (min)		Reaction product
	l-Amino acid	Products	Molecular mass (product name)
l-Homoserine	3.3	15.8	118.1 (2-oxo-4-hydroxybutyric acid)
l-Leucine	3.2	18.3	130.0 (2-oxo-4-methylvaleric acid)
l-Lysine	2.7	4.8	145.1 (2-oxo-6-aminohexanoic acid)
l-Glutamine	3.3	15.1	145.1 (2-oxo-4-carbamoylbutanoic acid)
l-Arginine	2.3	3.3	173.1 (2-oxo-5-guanidinopentanoic acid)
l-Glutamic acid-Na	14.6	18.5	190.0 (2-oxoglutaric acid)
l-Citrulline	3.2	14.9	174.0 (2-oxo-5-ureidovaleric acid)
		15.8	174.0 (pyrrolidine-1-carbamyl-2-hydroxy-2-carboxylic acid)
l-Phenylaranine	3.5	24.9	164.1 (2-oxo-3-phenylpropionic acid)
l-Histidine	3.0	17.9	154.3 (2-oxo-4-imidazolepropionic acid)
*N*ε**-Acetyl-l-lysine	3.2	15.5	187.1 (2-oxo-6-acetylaminohexanoic acid)
